# Prevalence of Anemia in Chronic Kidney Disease in the United States

**DOI:** 10.1371/journal.pone.0084943

**Published:** 2014-01-02

**Authors:** Melissa E. Stauffer, Tao Fan

**Affiliations:** 1 SCRIBCO, Effort, Pennsylvania, United States of America; 2 Merck & Co., Inc., Whitehouse Station, New Jersey, United States of America; Institut national de la santé et de la recherche médicale (INSERM), France

## Abstract

Anemia is one of the many complications of chronic kidney disease (CKD). However, the current prevalence of anemia in CKD patients in the United States is not known. Data from the National Health and Nutrition Examination Survey (NHANES) in 2007–2008 and 2009–2010 were used to determine the prevalence of anemia in subjects with CKD. The analysis was limited to adults aged >18 who participated in both the interview and exam components of the survey. Three outcomes were assessed: the prevalence of CKD, the prevalence of anemia in subjects with CKD, and the self-reported treatment of anemia. CKD was classified into 5 stages based on the glomerular filtration rate and evidence of kidney damage, in accordance with the guidelines of the National Kidney Foundation. Anemia was defined as serum hemoglobin levels ≤12 g/dL in women and ≤13 g/dL in men. We found that an estimated 14.0% of the US adult population had CKD in 2007–2010. Anemia was twice as prevalent in people with CKD (15.4%) as in the general population (7.6%). The prevalence of anemia increased with stage of CKD, from 8.4% at stage 1 to 53.4% at stage 5. A total of 22.8% of CKD patients with anemia reported being treated for anemia within the previous 3 months–14.6% of patients at CKD stages 1–2 and 26.4% of patients at stages 3–4. These results update our knowledge of the prevalence and treatment of anemia in CKD in the United States.

## Introduction

The kidneys function as filters of the blood, removing waste products and controlling the balance of fluid and electrolytes. Filtration occurs via bundles of capillaries called glomeruli (singular, glomerulus). A reduction in the glomerular filtration rate (GFR ) to <60 mL/min/1.73 m^2^ indicates chronic kidney disease (CKD), as do structural or functional renal abnormalities, which may be present in people with normal GFR [1]. Cross-sectional estimates of the prevalence of CKD in the United States range from 1.5% to 15.6% [2].

One of the lesser known functions of the kidneys is the production of erythropoietin, a signaling molecule that stimulates red blood cell production, in response to decreased oxygen levels in the blood. Any disruption of this process, e.g., secondary to a functional abnormality due to CKD, has the potential to produce anemia, a condition in which the number of circulating red blood cells, and therefore the level of hemoglobin, is lower than normal [3].

Other possible causes of anemia in CKD include iron deficiency, inflammation, and the accumulation of uremic toxins [3], [4]. Thus, the abnormal composition of blood or urine is an additional indicator of kidney damage.

Anemia in CKD is associated with cognitive impairment, sleep disturbances, CKD progression, cardiovascular comorbidities, and higher mortality [3], [5]–[7]. Direct healthcare costs are higher in CKD patients with anemia than in those without [7], and quality of life issues (e.g., fatigue, reduced productivity) are common [3], [5]. The prevalence of anemia (with or without CKD) increases with age [8], [9], which means that, as the US population ages, the number of people affected by anemia in CKD will also increase.

Available population-based determinations of the prevalence of anemia in CKD are becoming dated, with many studies referring back to the National Health and Nutrition Examination Survey (NHANES) III, which ended in 1994 [8], [10]–[12]. The most recent studies include NHANES data up to 2006 [8], [13], but one was limited to adults over age 64 with advanced CKD [13] and the other used a GFR classification not directly comparable to that of most other studies [8]. This analysis assessed the prevalence of anemia in CKD in the adult (>18 years of age) US population during 2007–2010 using the GFR categories specified by the National Kidney Foundation.

## Methods

### Study Design and Data Source

This was an analysis of cross-sectional data from the NHANES in 2007–2008 and 2009–2010. The NHANES is a biennial national survey that assesses the physical health of the non-institutionalized civilian population in the United States (www.cdc.gov/nchs/nhanes.htm). It is carried out and overseen by the National Center for Health Statistics, whose institutional review board approves each survey cycle.

### Study Sample

The study sample comprised adult subjects aged >18 years who participated in both the interview and examination components of NHANES. Participants provided written informed consent at the time of the survey.

### Study Variables

Glomerular filtration rate (GFR) was calculated using the formula GFR = 175×(S_Cr_)^−1.154^×(age)^−0.203^×(0.742 if female)×(1.210 if African American), where S_Cr_ is serum creatinine in mg/dL and age is expressed in years [14]. GFR units are mL/min/1.73 m^2^. CKD was classified into five stages defined by the GFR and/or evidence of kidney damage, as recommended by the National Kidney Foundation [14] and shown in Table 1. Evidence of kidney damage was derived from the presence of albuminuria, which is defined as a urinary albumin-to-creatinine ratio (ACR) ≥30 mg/g [14]. The NHANES 2009–2010 data set provided a variable for the urinary ACR (URDACT), but the 2007–2008 data set did not. Therefore, the urinary ACR was calculated for both survey periods from the variables for urinary albumin and creatinine as follows: ACR (mg/g) = urinary albumin (mg/dL)/urinary creatinine (g/dL). Stage of CKD was not calculated if data for either GFR or ACR were missing, except when GFR values alone indicated CKD of stages 3–5. Anemia was defined as serum hemoglobin levels ≤12 g/dL in women and ≤13 g/dL in men, as recommended by the National Anemia Action Council and the World Health Organization [15], [16].

**Table 1:** Classification of stage of chronic kidney disease.

Stage	Criteria
1	GFR ≥90 plus evidence of kidney damage
2	GFR 60–89 plus evidence of kidney damage
3	GFR 30–59
4	GFR 15–29
5	GFR <15

Stages of CKD were defined in accordance with the recommendations of the National Kidney Foundation. [14].

### Statistical Analysis

All analyses were performed in Stata MP v. 12.1 (StataCorp, College Station, TX). Four-year sample weights were calculated for the combined survey cycles as described in the NHANES analytic guidelines [17]. The sample weights were applied to calculate the proportions of subjects with CKD, anemia, and treatment for anemia. Standard errors obtained by Taylor series linearization were used to obtain 95% confidence intervals. Each outcome was determined for all subjects and by stage of CKD. The ‘subpopulation’ option in Stata was used for analyses of subgroups. Subjects with no calculated stage of CKD (N = 977; 8.1%) were classified as not having CKD. The prevalence of anemia was determined in all subjects and then separately in subjects with and without CKD. Treatment for anemia was assessed only in subjects with anemia (and CKD). Prevalence values were converted to projections of the respective numbers in the US population based on the 2000 US census.

## Results

### Participants

A total of 12,077 adults participated in the interview and examination components of the NHANES surveys in 2007–2008 and 2009–2010.

### Prevalence of CKD

The prevalence of CKD was 14.0% (Table 2). This represents an estimated 31.4 million people in the US population. About half (48.4%) of the people with CKD were at stage 3. A similar proportion (46.9%) was in stages 1–2 combined. Only 4.7% were in stages 4–5.

**Table 2:** Prevalence of chronic kidney disease.

	N	Weighted percentage (95% CI)	95% CI	Projected number in US
With CKD	2,125	14.0	13.2–15.0	31.4×10^6^
Stage 1	507	3.1	2.7–3.6	6.8×10^6^
Stage 2	489	3.4	2.9–3.9	7.4×10^6^
Stage 3	1,028	7.0	6.2–7.9	16×10^6^
Stage 4	70	0.4	0.3–0.5	0.9×10^6^
Stage 5	31	0.1	0.1–0.2	0.3×10^6^
Without CKD	9,952	86.0	85.0–86.9	190×10^6^

Percentages reflect the prevalence of each stage of CKD, survey weighted to the US population. The analysis was limited to subjects aged >18 with data from both the exam and interview components of the survey (N = 12,077).

### Prevalence of Anemia in CKD

The prevalence of anemia in the combined survey population, regardless of CKD, was 7.6% (result not shown). The prevalence of anemia was 15.4% in people with CKD (Table 3). This represents an estimated 4.8 million people. The prevalence of anemia increased with stage of CKD, from 8.4% at stage 1 to 53.4% at stage 5. The prevalence of anemia in people without CKD was 6.3%.

**Table 3:** Prevalence of anemia.

	N	Weightedpercentage	95% CI	Projected number in US
With CKD	410	15.4	13.1–18.2	4.8×10^6^
Stage 1	57	8.4	5.5–12.4	0.6×10^6^
Stage 2	68	12.2	9.2–16.0	0.9×10^6^
Stage 3	231	17.4	13.7–21.8	2.7×10^6^
Stage 4	37	50.3	37.2–63.4	0.5×10^6^
Stage 5	17	53.4	34.1–71.7	0.2×10^6^
Without CKD	729	6.3	5.3–7.4	11×10^6^

The percentages reflect the prevalence of anemia, survey weighted to the US population. The analysis by stage of CKD was limited to subjects with CKD as defined in the Methods section (N = 2,125); data for anemia were missing for a total of 7 CKD patients (<1%), all in stages 1–3. Prevalence of anemia in subjects without CKD was determined in 9,269 subjects with non-missing data on anemia status.

### Treatment of Anemia

A total of 22.8% of CKD patients with anemia reported being treated for it (Table 4). Treatment frequencies were similar at stages 1 and 2 (12.1% and 16.2%; P = 0.57; weighted mean 14.6%) and stages 3 and 4 (26.5% and 20.7%; P = 0.76; weighted mean 26.4%).

**Table 4:** Self-reported treatment of anemia in chronic kidney disease.

Stage of CKD	N	Weightedpercentage	95% CI
1	8	12.1	5.4–25.0
2	9	16.2	7.0–33.3
3	59	26.5	21.2–32.6
4	8	20.7	8.8–41.2
5	6	43.0	17.1–73.4
Total	90	22.8	18.5–27.7

The weighted percentages reflect the frequency of treatment of anemia among people within each stage of CKD, survey weighted to the US population. The analysis was limited to subjects with anemia (N = 410).

## Discussion

In our analysis of NHANES data from 2007–2010, an estimated 14.0% of the US population had CKD. There are several indications that this value represents a slightly increasing prevalence of CKD in the United States. The first is a comparison with an analysis of the NHANES III (1988–1994), in which the prevalence of CKD was 11.0% [18]. Secondly, the unweighted prevalence of CKD in the NHANES 1999–2004 surveys was reported to be 16.8% [19], slightly lower than the unweighted prevalence in the current study (17.6%). Thirdly, Coresh et al. reported prevalence values of 9.4–13.1% for stages 1–4 of CKD in various analyses covering NHANES III through 2004 [20], [21]. Our analysis of stages 1–4 in years 2007–2010 yielded an estimated prevalence of 13.9%. Finally, reports on the Kidney Early Evaluation Program (KEEP) cohort of patients at high risk for CKD indicate that 15.6% of the cohort had GFRs corresponding to CKD stages 3–5 in 2000–2001 [22], whereas 16.8% met this criterion over the period 2000–2009 [23].

In our analysis, anemia was estimated to be present in 15.4% of people with any stage of CKD. The prevalence of anemia increased with stage of CKD, from 8.4% at stage 1 to 53.4% at stage 5. A similar trend has been reported by several other authors [11], [24], [25], consistent with the known pathogenesis of CKD (i.e., that erythropoietin production decreases as kidney function worsens). Fishbane et al. found high rates of iron deficiency in adult men (57.8 to 58.8%) and women (69.9 to 72.8%) with CKD stages 3–5 in the NHANES III and 1999–2004 surveys [12], indicating that anemia in higher stage CKD may have multiple causes.

Other studies of the prevalence of anemia in CKD focused on older adults (>64 years of age). According to Stevens et al., 29.9% of KEEP 2000–08 participants and 19.9% of NHANES 1999–2006 subjects in CKD stages 3–5 and over age 64 had comorbid anemia, defined as hemoglobin levels <13.5 g/dL for men and <12 g/dL for women [13]. For comparison, the prevalence of anemia in stage 3–5 CKD patients aged >64 in the 2007–2010 NHANES surveys was 24.4%. A higher prevalence of anemia (64.9%; defined as hemoglobin <13 g/dL for men, <12 g/dL for women) was observed in US nursing home residents aged >64 with CKD stages 3–5 [25].

Treatments for anemia include iron supplementation and erythropoietin stimulating agents. Reported treatment rates are typically low (10–15%), regardless of the source of data. In an analysis of the National Ambulatory Medical Care Survey (1996–2002), just 10% of outpatient visits for anemia management in CKD resulted in prescription of medication for anemia [26]. In an online survey conducted in 2006, just 15% of 376 family medicine and internal medicine trainees in the United States reported that they would initiate treatment for anemia in CKD at hemoglobin levels <11 g/dL [27]. In our analysis of NHANES 2007–2010, 22.8% of CKD patients with anemia reported being treated for anemia within the previous 3 months.

Insight into the usual timing and types of treatments received (at least among older men) can be gleaned from a study of the Veterans Health Administration database by Lawler et al. [28]. Among 89,000 patients with anemia and CKD (96.7% male, median age 77.5), 7.1% initiated erythropoietin stimulating agents within a year of diagnosis of anemia, while 30.8% initiated iron therapy and 16.5% used blood transfusions in the same time span. The median time to erythropoietin stimulating agent initiation was 138 days.

CKD prevalence values can vary markedly depending on how GFR is estimated [23], [29]. We chose to use the definitions of CKD and GFR established by the National Kidney Foundation to maximize the comparability of our results with previous NHANES and other studies that used these same definitions. We note that a new method for estimating GFR has been shown to be slightly more accurate in classifying patients by stage of CKD [23]. In addition, hemoglobin levels may vary not only by sex but also by age, pregnancy, altitude and smoking status [30]. We used the definitions of anemia given by the National Anemia Action Council and the World Health Organization for simplicity, but did not account for the variables listed above. Our analysis of treatment for anemia was limited by the fact that NHANES data on the various treatments for anemia (e.g., ESAs, iron) were sparse.

Several elements of the study design may have skewed our estimation of the prevalence of CKD. First, inclusion of subject with missing data on GFR and/or ACR likely resulted in underestimation of the full number of people with CKD. On the other hand, albuminuria was assessed with a single spot urine test in NHANES and may have been a temporary condition, resulting in overestimation of the number of subjects at CKD stages 1–2. Lastly, as described in other NHANES-based analyses of CKD, “nonparticipation rates caused by illness relating to having a very low GFR are expected to be particularly high in [stage 5], severely downwardly biasing these prevalence estimates” [18].

In summary, CKD prevalence appears to be increasing in the US adult population. In 2007–2010, anemia was present in approximately 15% of CKD patients and was more frequent at higher stages of CKD. Relatively few CKD patients with anemia were being treated for anemia.

## References

[1] National Kidney Foundation (2002) K/DOQI Clinical Practice Guidelines for Chronic Kidney Disease: Evaluation, Classification and Stratification. Am J Kidney Dis 39: S1–S266. 11904577

[2] Zhang QL, Rothenbacher D (2008) Prevalence of chronic kidney disease in population-based studies: systematic review. BMC Public Health 8: 117. 18405348 10.1186/1471-2458-8-117 PMC2377260

[3] Smith RE, Jr. (2010) The clinical and economic burden of anemia. Am J Manag Care 16 Suppl Issues: S59–66. 20297873

[4] National Kidney Foundation (2006) KDOQI Clinical Practice Guidelines and Clinical Practice Recommendations for Anemia in Chronic Kidney Disease. Am J Kidney Dis 47: S11–145. 16678659 10.1053/j.ajkd.2006.03.010

[5] Mehdi U, Toto RD (2009) Anemia, diabetes, and chronic kidney disease. Diabetes Care 32: 1320–1326. 19564475 10.2337/dc08-0779 PMC2699743

[6] Herzog CA, Muster HA, Li S, Collins AJ (2004) Impact of congestive heart failure, chronic kidney disease, and anemia on survival in the Medicare population. J Card Fail 10: 467–472. 15599836 10.1016/j.cardfail.2004.03.003

[7] van Nooten FE, Green J, Brown R, Finkelstein FO, Wish J (2010) Burden of illness for patients with non-dialysis chronic kidney disease and anemia in the United States: review of the literature. J Med Econ 13: 241–256. 20438399 10.3111/13696998.2010.484307

[8] Bowling CB, Inker LA, Gutierrez OM, Allman RM, Warnock DG, et al (2011) Age-specific associations of reduced estimated glomerular filtration rate with concurrent chronic kidney disease complications. Clin J Am Soc Nephrol 6: 2822–2828. 22034504 10.2215/CJN.06770711 PMC3255373

[9] Nissenson AR, Wade S, Goodnough T, Knight K, Dubois RW (2005) Economic burden of anemia in an insured population. J Manag Care Pharm 11: 565–574. 16137214 10.18553/jmcp.2005.11.7.565 PMC10437330

[10] Hsu CY, McCulloch CE, Curhan GC (2002) Epidemiology of anemia associated with chronic renal insufficiency among adults in the United States: results from the Third National Health and Nutrition Examination Survey. J Am Soc Nephrol 13: 504–510. 11805181 10.1681/ASN.V132504

[11] Clase CM, Kiberd BA, Garg AX (2007) Relationship between glomerular filtration rate and the prevalence of metabolic abnormalities: results from the Third National Health and Nutrition Examination Survey (NHANES III). Nephron Clin Pract 105: c178–184. 17347576 10.1159/000100489

[12] Fishbane S, Pollack S, Feldman HI, Joffe MM (2009) Iron indices in chronic kidney disease in the National Health and Nutritional Examination Survey 1988–2004. Clin J Am Soc Nephrol 4: 57–61. 18987297 10.2215/CJN.01670408 PMC2615715

[13] Stevens LA, Li S, Wang C, Huang C, Becker BN, et al (2010) Prevalence of CKD and comorbid illness in elderly patients in the United States: results from the Kidney Early Evaluation Program (KEEP). Am J Kidney Dis 55: S23–33. 20172445 10.1053/j.ajkd.2009.09.035 PMC4574484

[14] National Kidney Foundation. Frequently Asked Questions about GFR Estimates. http://www.kidney.org/professionals/kls/pdf/12-10-4004_KBB_FAQs_AboutGFR-1.pdf. Accessed August 1, 2012.

[15] National Anemia Action Council. Anemia & Kidney Disease. http://www.anemia.org/patients/information-handouts/kidney-disease/?handout=kidney-disease%2F. Accessed August 1, 2012.

[16] World Health Organization (2001) Iron Deficiency Anaemia: Assessment, Prevention, and Control. Geneva, Switzerland. Available at: http://www.who.int/nutrition/publications/micronutrients/anaemia_iron_deficiency/WHO_NHD_01.3/en/index.html.

[17] Centers for Disease Control and Prevention. (2012) National Health and Nutrition Examination Survey Analytic and Reporting Guidelines. http://www.cdc.gov/nchs/nhanes/nhanes2003-2004/analytical_guidelines.htm. Accessed October 18, 2012.

[18] Coresh J, Astor BC, Greene T, Eknoyan G, Levey AS (2003) Prevalence of chronic kidney disease and decreased kidney function in the adult US population: Third National Health and Nutrition Examination Survey. Am J Kidney Dis 41: 1–12. 12500213 10.1053/ajkd.2003.50007

[19] Centers for Disease Control and Prevention (2007) Prevalence of Chronic Kidney Disease and Associated Risk Factors – United States, 1999–2004. Morbidity and Mortality Weekly Report 58: 161–165. 17332726

[20] Coresh J, Byrd-Holt D, Astor BC, Briggs JP, Eggers PW, et al (2005) Chronic kidney disease awareness, prevalence, and trends among U.S. adults, 1999 to 2000. J Am Soc Nephrol 16: 180–188. 15563563 10.1681/ASN.2004070539

[21] Coresh J, Selvin E, Stevens LA, Manzi J, Kusek JW, et al (2007) Prevalence of chronic kidney disease in the United States. JAMA 298: 2038–2047. 17986697 10.1001/jama.298.17.2038

[22] Brown WW, Peters RM, Ohmit SE, Keane WF, Collins A, et al (2003) Early detection of kidney disease in community settings: the Kidney Early Evaluation Program (KEEP). Am J Kidney Dis 42: 22–35. 12830453 10.1016/s0272-6386(03)00405-0

[23] Stevens LA, Li S, Kurella Tamura M, Chen SC, Vassalotti JA, et al (2011) Comparison of the CKD Epidemiology Collaboration (CKD-EPI) and Modification of Diet in Renal Disease (MDRD) study equations: risk factors for and complications of CKD and mortality in the Kidney Early Evaluation Program (KEEP). Am J Kidney Dis 57: S9–16. 21338849 10.1053/j.ajkd.2010.11.007 PMC3298760

[24] Moossavi S, Freedman BI (2009) Treating anemia with erythropoiesis-stimulating agents: effects on quality of life. Arch Intern Med 169: 1100–1101. 19546408 10.1001/archinternmed.2009.159

[25] Robinson B, Artz AS, Culleton B, Critchlow C, Sciarra A, et al (2007) Prevalence of anemia in the nursing home: contribution of chronic kidney disease. J Am Geriatr Soc 55: 1566–1570. 17727646 10.1111/j.1532-5415.2007.01389.x

[26] Rasu RS, Manley HJ, Crawford T, Balkrishnan R (2007) Undertreatment of anemia in patients with chronic kidney disease in the United States: analysis of national outpatient survey data. Clin Ther 29: 1524–1534. 17825703 10.1016/j.clinthera.2007.07.016

[27] Lenz O, Fornoni A (2006) Chronic kidney disease care delivered by US family medicine and internal medicine trainees: results from an online survey. BMC Med 4: 30. 17164005 10.1186/1741-7015-4-30 PMC1713248

[28] Lawler EV, Gagnon DR, Fink J, Seliger S, Fonda J, et al (2010) Initiation of anaemia management in patients with chronic kidney disease not on dialysis in the Veterans Health Administration. Nephrol Dial Transplant 25: 2237–2244. 20083469 10.1093/ndt/gfp758

[29] Snyder JJ, Foley RN, Collins AJ (2009) Prevalence of CKD in the United States: a sensitivity analysis using the National Health and Nutrition Examination Survey (NHANES) 1999–2004. Am J Kidney Dis 53: 218–228. 18950914 10.1053/j.ajkd.2008.07.034 PMC2664624

[30] Agarwal AK (2006) Practical approach to the diagnosis and treatment of anemia associated with CKD in elderly. J Am Med Dir Assoc 7: S7–S12; quiz S17–21. 10.1016/j.jamda.2006.09.005 17098634

